# Relationships

**Published:** 1892-02

**Authors:** H. H. Harrison


### Relationships.

BY H. H. HARRISON.

Read before the Ohio State Society, December 2d, 1891.

How wonderful is the law that binds together all created things and yet in such a way that it does not destroy the identity of any thing.

The earth and atmosphere contain the elements which enter into the formation of all organic or inorganic creations. The law which holds together and controls organized matter is known as life, while the law that controls matter without life is known as accretion.

The higher principles—life and accretion—which have the power to draw and select for their own growth and development the various elements in the form of atoms, possess an inherent quality or characteristic that, to say the least, is phenomenal, and probably will remain so to the end of time.

To observe the law of relationship and see how one order of creation is dependent upon another we will select the highest type of created objects—man.

The Bible says: "Man is fearfully and wonderfully made," and the anatomist and physiologist must necessarily agree to the Bible statement. And yet what is man when the life is removed but a bundle of earthly atoms, water and gas, selected and brought together by the action of that inscrutable law of life. How this life-principle can select and appropriate the different elements must always be answered by—I don't know. But as to the elements themselves we do know.

We do know, first, that all the supply for the human body comes through the vegetable creations, drawn up from the earth and imbibed from the atmosphere in the proper proportion to suit the requirements of the body.

Now, when the power that selects these elements dies, or leaves the body, it is but natural that they go back to their original state. This being true, there must be a law governing this process with active agents to perform the work.

This we know by experience to be true and the same elements that entered into our body may come up again through the vegetable life and assist in the formation of another human body. And so the elements are going around in a circle as the earth, the planets and stars go around in their circular orbit.

These elements that go to form the body, as before stated, are of the quality to establish a well-formed, healthful organism, but do they always do so? If not, why?

By the ingenuity of man himself unwarranted selections are made, and that which nature designed to be used is laid aside and the body fails to get the proper proportions; as a result the organic structure of man is weakened, and as a final result, disease follows. The term, disease itself, is suggestive of this, for it means not at ease, showing the improper relationship of matter.

When our bodies are not properly nourished, when poison is introduced, or when wounded, nature sets up a restorative process, and pain is the result. Pain, then is an indication of improper relationship and is not found in the body where physiological law obtains, save in one state, which is that of parturition.

We all know that the body is subject to pathological conditions and all brought about by faulty relationship. The diseased state of the teeth and associate parts is due to the same great law.

We are aware that in the etiology of disease we have the predisposing and exciting causes. But when asked for the cause of caries, or rather what it is, our text books come to our relief and say: "Caries is a chemical decomposition of the earthy salts of the affected parts;" concise in one respect, and very vague in another.

The predisposing cause is left out, and yet without it we would never have decay of the teeth at all. We can say truthfully caries is the disease, and the cause is defective nutrition.

Let us get at it in another way and say caries, the disease, is caused by imperfect assimilation, and the operation of destruction is a natural law removing dead or effete matter from the living body. The first is a violation of natural law, while the second is a fulfillment of the same law, and is naught but the tearing-down process, mentioned before, when all dead tissue is dissolved and returns to its original state—" Earth to earth and dust to dust."

The great aim of dentists in the past has been to prevent the secondary cause of caries, leaving the primary cause severely alone. And while the action is perfectly justifiable and commendable in arresting the progress of disease, yet we are not doing all we should or could, in urging our patients to fortify themselves against the violation of this great law of nutrition.

General practitioners of medicine have not the opportunity to observe the great destruction brought about by the want of better nourishment of the bony system that dentists have. And hence, we ought to speak with no uncertain sound.

Where we find teeth weak and enfeebled, there is also a correspondingly defective development of all the bones of the body wherein is developed as a result, spinal curvature, crooked limbs and a general nervous disturbance.

It is very much the same with the human body as it is in architecture. If the foundation and frame-work be badly executed and made out of poor material no amount of elaboration by paint or finely chiseled sculpture or ornamentation can make it a good and durable building. Then, when we are faithfully combating the disease of caries by the best efforts and most approved remedies let us not overlook that principle which permits this disease to exist. By doing so we may be philanthropists to the coming generation, and dentistry will have scored a high mark in the history of science that will go down along the coming ages with distinction and honor. "So mote it be."

